# A case report of eosinophilic granulomatosis with polyangiitis in children with cerebella infarction as the first symptom and literature review

**DOI:** 10.3389/fimmu.2025.1682325

**Published:** 2025-11-20

**Authors:** Dingyun Chen, Xiaoliang He, Yang Shen, Yutong Gao, Denghuan Chen, Shouwei Hang, Xinrong Wang, Na Li, Daliang Xu

**Affiliations:** Department of Rheumatology, Anhui Provincial Children’s Hospital, Hefei, Anhui, China

**Keywords:** eosinophilic granulomatous polyangiitis, cerebella infarction, eosinophils, children, central nervous system

## Abstract

**Objective:**

This case report presents a pediatric case of eosinophilic granulomatosis with polyangiitis (EGPA) presenting with cerebella infarction as the initial symptom. The study aims to summarize the clinical features and treatment advancements of this condition, enhancing clinicians ‘understanding and reducing misdiagnosis and missed diagnosis.

**Methods:**

A 9-year-old male patient admitted to the Rheumatology Department of Anhui Provincial Children’s Hospital was evaluated for “Dizziness, vomiting for one day, and consciousness impairment for half a day.” The patient had recurrent bilateral lower limb rashes and asthma attacks over the past year. Blood tests revealed elevated eosinophil levels and IgE antibodies, while bone marrow cytology showed increased eosinophil counts. Brain MRI demonstrated cerebral infarction, herniation, and suspected thrombosis, with skin biopsy confirming vasculitis characteristics. Through retrospective analysis of clinical data and literature review, this study comprehensively summarizes EGPA’s clinical features and treatment progress.

**Results:**

The patient presented with cerebella infarction as the initial symptom, accompanied by central nervous system involvement, skin manifestations, hematological disorders, and vasculitis. With a history of asthma attacks, eosinophil counts during hospitalization peaked at 4.9×10^9/L (compared to baseline levels>1×10^9/L). After thorough evaluation for infections, malignancies, diffuse connective tissue diseases, immunodeficiency disorders, and inherited metabolic disorders, EGPA was confirmed. Treatment included anti-inflammatory steroids, cyclophosphamide (CTX) induction therapy, anticoagulation, followed by mycophenolate mofetil (MMF) maintenance at therapeutic doses, supplemented with rituximab. Current follow-up shows normalized eosinophil counts, restored muscle strength, resolution of skin rashes without recurrence, and favorable clinical response.

**Conclusion:**

EGPA presents diverse clinical features. Cerebella infarction as the first neurological manifestation in children is rare. Eosinophilia serves as a characteristic feature. When pediatric asthma patients exhibit neurological symptoms, EGPA should be considered. For cases with central nervous system involvement, combined steroid therapy with cyclophosphamide pulse induction proves effective.

## 1 Introduction

Eosinophilic Granulomatous Vasculitis (EGPA) is a rare autoimmune disorder that can affect multiple systems throughout the body. It is characterized by increased eosinophil counts, tissue infiltration, and necrotizing granulomatous inflammation in small and medium blood vessels. Since it primarily affects the respiratory tract and lungs, most patients initially present with wheezing episodes or sinusitis symptoms, often leading to misdiagnosis as bronchial asthma by respiratory specialists. As the disease progresses, it may involve multiple systems including the skin, sinuses, lungs, nervous system, heart, gastrointestinal tract, and kidneys. Notably, central nervous system involvement has been reported both domestically and internationally (1–3), though cases presenting with CNS involvement as the initial presentation are relatively rare in children. This study systematically analyzes a pediatric EGPA case involving neurological involvement treated in our department, combining clinical data with literature reviews to evaluate its clinical features, treatment strategies, and prognosis. The aim is to enhance understanding of this condition to facilitate early diagnosis and improve patient outcomes.

## 2 Case presentation

The patient is a 9-year-old boy with a history of recurrent red rashes on both lower limbs accompanied by significant itching for one year, and an asthma attack in the past 8 months. He was admitted to our hospital on August 31,2024, due to “dizziness, vomiting for 1 day, and impaired consciousness for half a day.” One day before admission, he experienced dizziness accompanied by nausea and vomiting while climbing stairs. The vomiting was non-projectile, with gastric contents as vomitus. A cranial CT scan at the local hospital revealed atlantoaxial subluxation, while an ECG showed sinus tachycardia and T-wave abnormalities. Blood tests indicated abnormally elevated eosinophil percentage (55.8%). Symptomatic treatments included mannitol for intracranial pressure reduction, cimetidine for antihistamine, methylprednisolone for anti-inflammatory, fluid replacement, and cervical collar fixation. Symptoms showed no improvement, with increased vomiting frequency. On the morning of admission, he exhibited altered consciousness manifested as drowsiness. A cranial MRI at our hospital confirmed large-area acute infarction of the right cerebellar hemisphere and cerebellar tonsillar herniation, leading to admission under the diagnosis of “cerebral infarction.” During the course of illness, the child showed poor mental state and appetite, but normal urination and defecation. Medical history revealed a month prior outdoor activities in lakes with local skin abrasions, but no head or neck trauma. The mother had recurrent embryonic arrest causing miscarriage during pregnancy. This child is her 8th pregnancy and 2nd delivery (Gravidity 8, Parity 2), born at full term with 2900g birth weight via natural delivery without resuscitation history.

**Figure 1:**
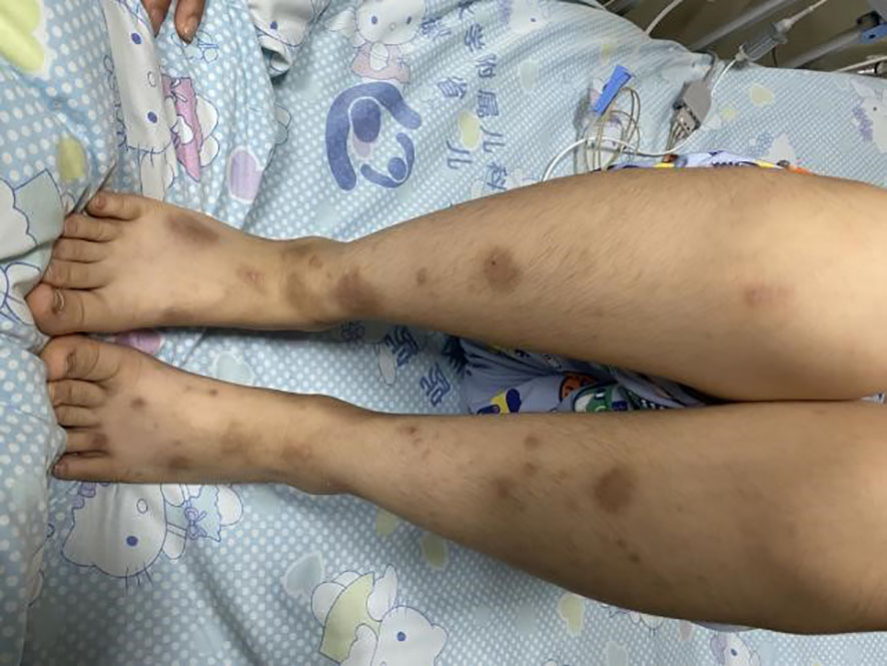
Scattered rash on both lower limbs.

Upon admission, the patient had a blood pressure of 127/83 mmHg, height 134 cm, weight 30 kg, and body surface area 1.06 m². The patient exhibited drowsiness, lethargy, and poor mental responsiveness. Scattered rashes were observed on both lower limbs (**Figure 1**), with a larger lesion measuring approximately 14×9 mm at the left ankle, showing central ulceration with crusting and localized swelling. Without oxygen therapy, the facial color remained normal. Bilateral pupils were equal in size and roundness, measuring about 4 mm in diameter, with sensitive light reflexes. The patient was secured with a cervical collar, maintaining a respiratory rate of approximately 29 breaths per minute. Bilateral lung sounds were coarse, with no significant dry or wet rales detected. Heart rate was 120 bpm with regular rhythm and audible heart sounds, and no murmurs were heard. The abdomen was flat and soft, with the liver located 1.5 cm below the costal margin and soft in consistency. The spleen was not palpable below the costal margin. All extremities were warm, with preserved abdominal reflexes, knee tendons, and Achilles tendon reflexes. Muscle strength was graded as grade III in both upper and lower limbs on the right side, and grade V in the left side. Muscle tone was normal, and bilateral Babinski signs were negative.

Laboratory findings upon admission revealed: Multiple complete blood counts showed elevated eosinophil ratios (up to 32.8% [reference range 0.5-9%]), with eosinophil counts reaching 4.90×10^9/L (normal range 0.04-0.74×10^9/L). Hemoglobin and platelet levels were within normal ranges. Total IgE levels were 4033.0IU/ml. Cytokine measurements included: interleukin-2 receptor 2846.4U/ml (220–720 U/ml), interleukin-eight 872.45pg/ml (below 60 pg/ml), interleukin-six 58.74pg/ml (below 7 pg/ml), while interleukin-1ß and interleukin-10 remained normal. Routine tests showed normal liver/kidney function, cardiac enzyme profiles, immunoglobulin levels, C-reactive protein, serum amyloid protein, erythrocyte sedimentation rate, rheumatoid factor, ferritin, coagulation panel, anti-neutrophil cytoplasmic antibodies (ANCA), antinuclear antibodies, antiphospholipid antibodies (both standard and non-standard), lupus antistroma, tumor markers, occult blood, proteinuria, whole-exome sequencing, and genetic screening for metabolic disorders. Pathogen tests—including peripatricidal pathogens, parasitic antibodies, cytomegalovirus antibodies, DNA testing, HIV antibodies, hepatitis B/C, tuberculin skin test, T-cell reactivity, blood cultures, and stool parasitology—were all negative.

**Figure 2:**
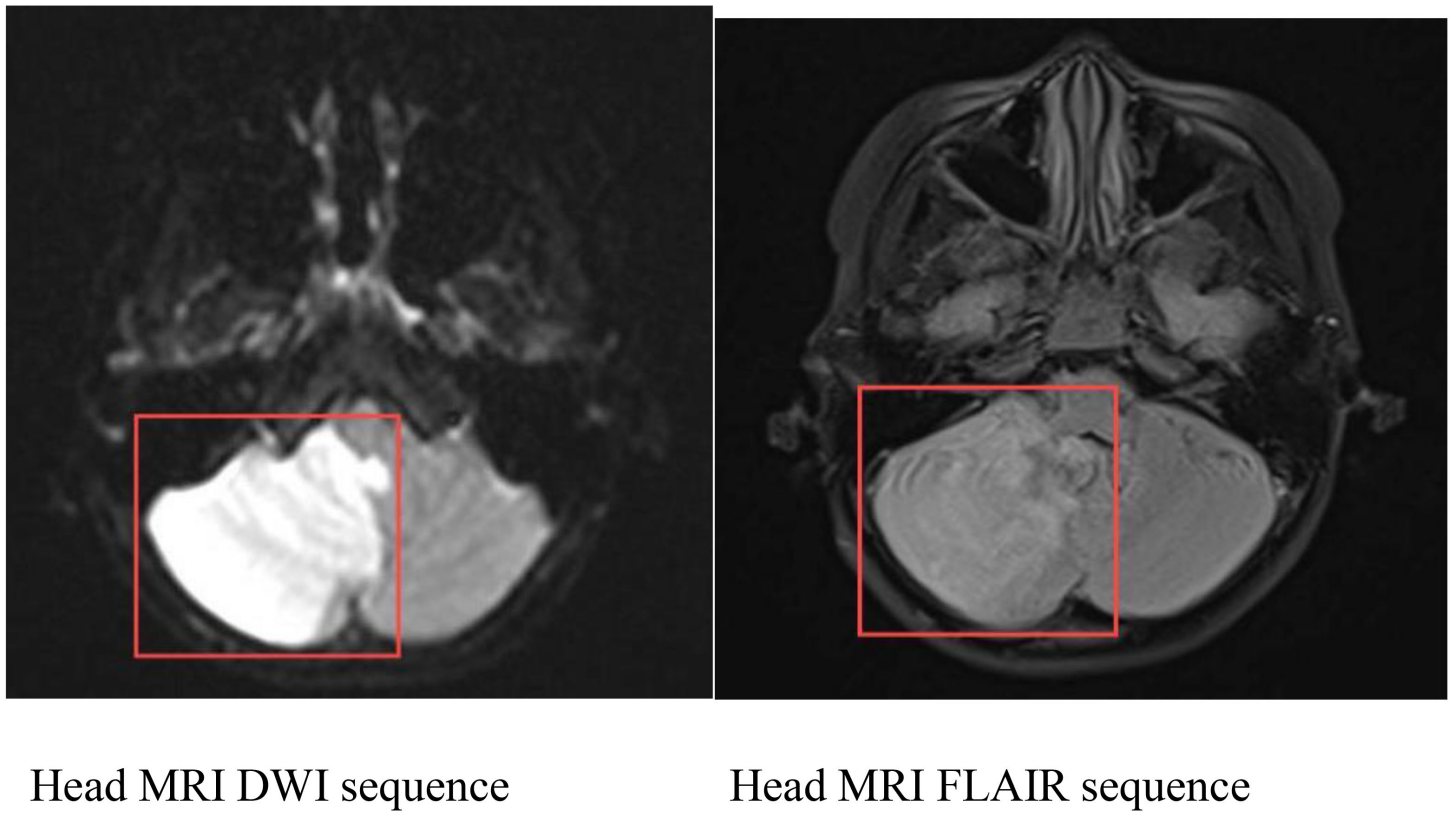
Cranial MRI: Abnormal signal in the right cerebellar hemisphere, considered as cerebral infarction; cerebellar tonsillar herniation; full supratentorial ventricle.

**Figure 3:**
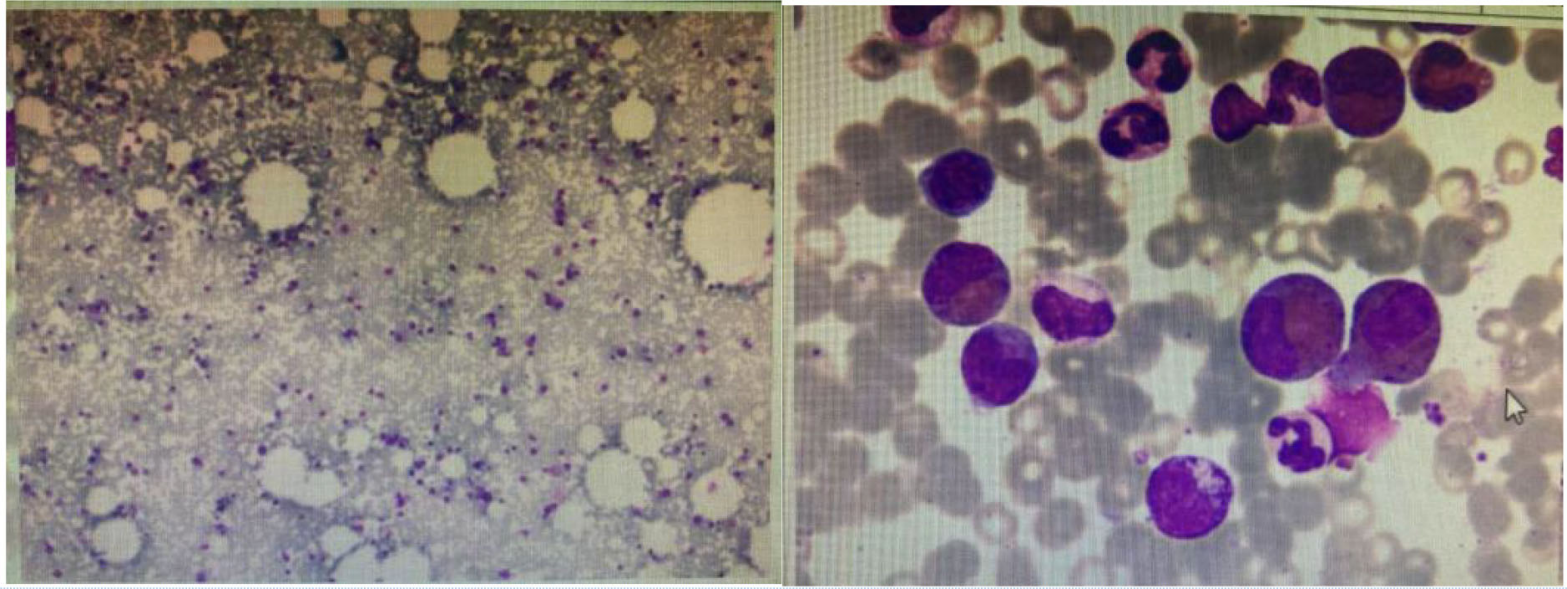
Shows an increased proportion of eosinophils in bone marrow cytology.

Head MRI at admission (September 1, 2024) (**Figure 2**): The cerebellum showed mild swelling with extensive long T1 and long T2 signals in the right cerebellar hemisphere. FLAIR and diffusion-weighted imaging (DWI) sequences demonstrated hyperintense signals. The supratentorial cerebellar tentorium was elevated, and the cerebellar base margin was slightly displaced downward beyond the level of the foramen magnum. The cerebral lobes showed normal gyri, while both lateral ventricles and the third ventricle were bulging. Brainstem structures remained normal. Diagnostic conclusions: Abnormal signals in the right cerebellar hemisphere suggest cerebral infarction; cerebellar tonsillar herniation; bulging supratentorial ventricles. Bone marrow cytology (**Figure 3**): Active bone marrow with marked granulocytic proliferation, erythrocytic proliferation, 61 megakaryocytes, and platelet aggregation. Elevated eosinophil percentage (25.5%). Pulmonary function tests revealed normal vital capacity (VC max) and forced vital capacity (FVC), but mild decreases in FEV1, FEV1/FVC, and peak expiratory flow (PEF). All expiratory flow rates were moderately reduced. Conclusion: The patient exhibited abnormal pulmonary ventilation with mild obstructive ventilatory dysfunction. High-resolution chest CT revealed no interstitial lung disease. Electromyography of the limbs, color Doppler ultrasound of the liver, gallbladder, pancreas, spleen, urinary tract, and retroperitoneal lymph nodes showed no abnormalities. No thickening or narrowing was observed in the walls of the limbs, bilateral neck arteries, bilateral iliac arteries, abdominal aorta, bilateral renal arteries, or inferior vena cava. To rule out spinal cord compression, injury, or space-occupying lesions, and to evaluate cerebral function, assess injury severity, and predict prognosis, a complete spinal MRI and EEG were performed. The spinal MRI results were normal, but the EEG showed abnormal background activity with bilateral posterior head slow-wave desynchronization, indicating potential cerebral dysfunction.

## 3 Treatment course

**Figure 4:**
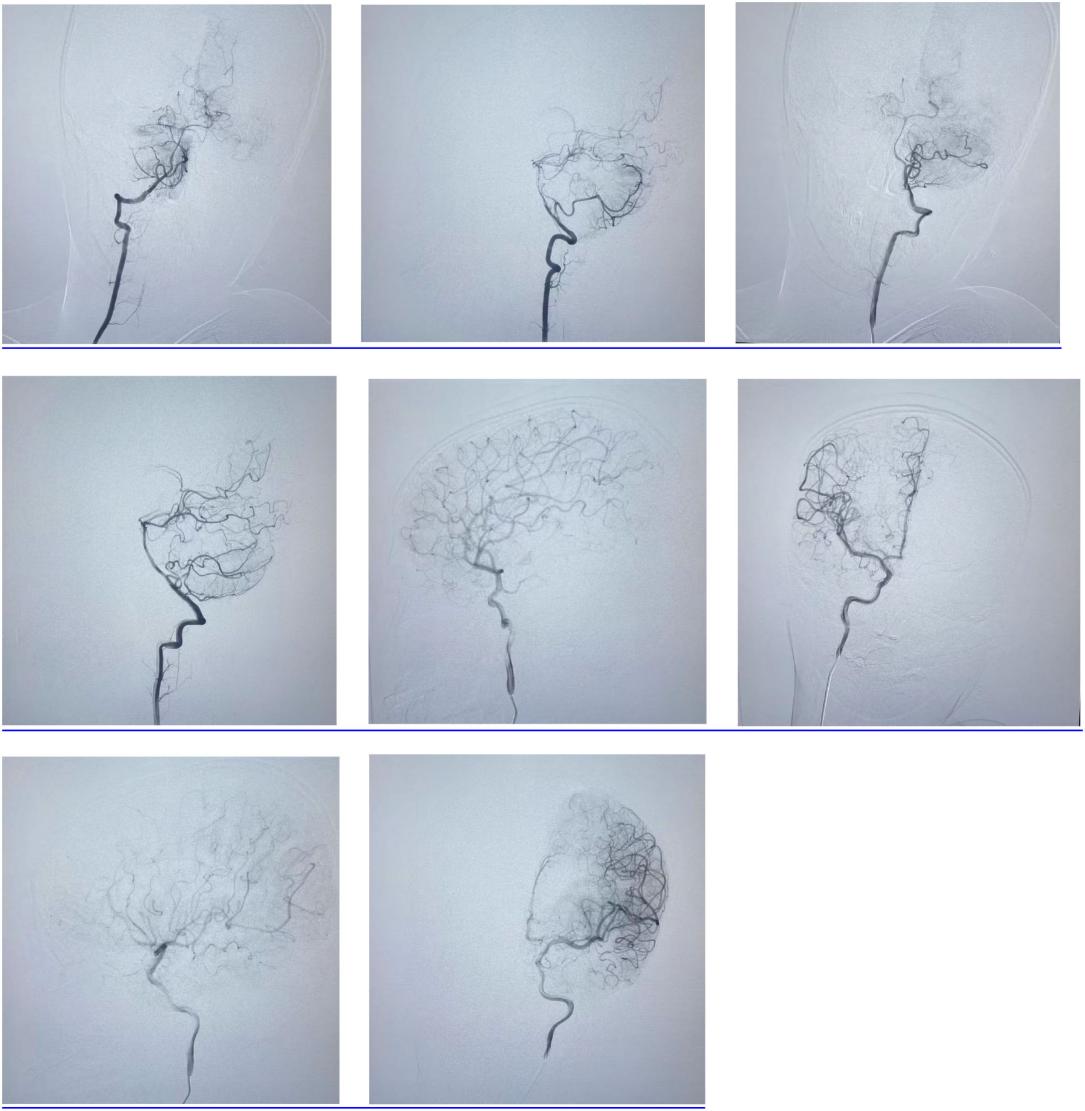
Cerebral angiography revealed patent visualization of both vertebral arteries, basilar artery, bilateral internal carotid arteries, and their branches, with no evidence of stenosis or occlusion.

After admission, the patient was first admitted to the Critical Care Unit of our hospital. Under general anesthesia in the emergency department, cerebral angiography was performed. The procedure revealed patent visualization of both vertebral arteries, basilar artery, bilateral internal carotid arteries, and their branches, with no evidence of stenosis or occlusion (**Figure 4**). Postoperatively, the patient was transferred to the ward for supportive care including mechanical ventilation, enteral nutrition, loratadine antihistamine therapy, mannitol-induced intracranial pressure reduction, and oxygen therapy. The patient’s condition gradually improved, with consciousness returning to normal and right limb muscle strength recovering to grade IV. However, follow-up tests showed persistently elevated eosinophil counts and proportions. On September 7th, a blood test revealed white blood cell count at 14.95×10^9/L with 32.8% eosinophil percentage and eosinophil count at 4.9×10^9/L. The rheumatology department requested a consultation to determine further diagnostic and therapeutic approaches.

**Figure 5:**
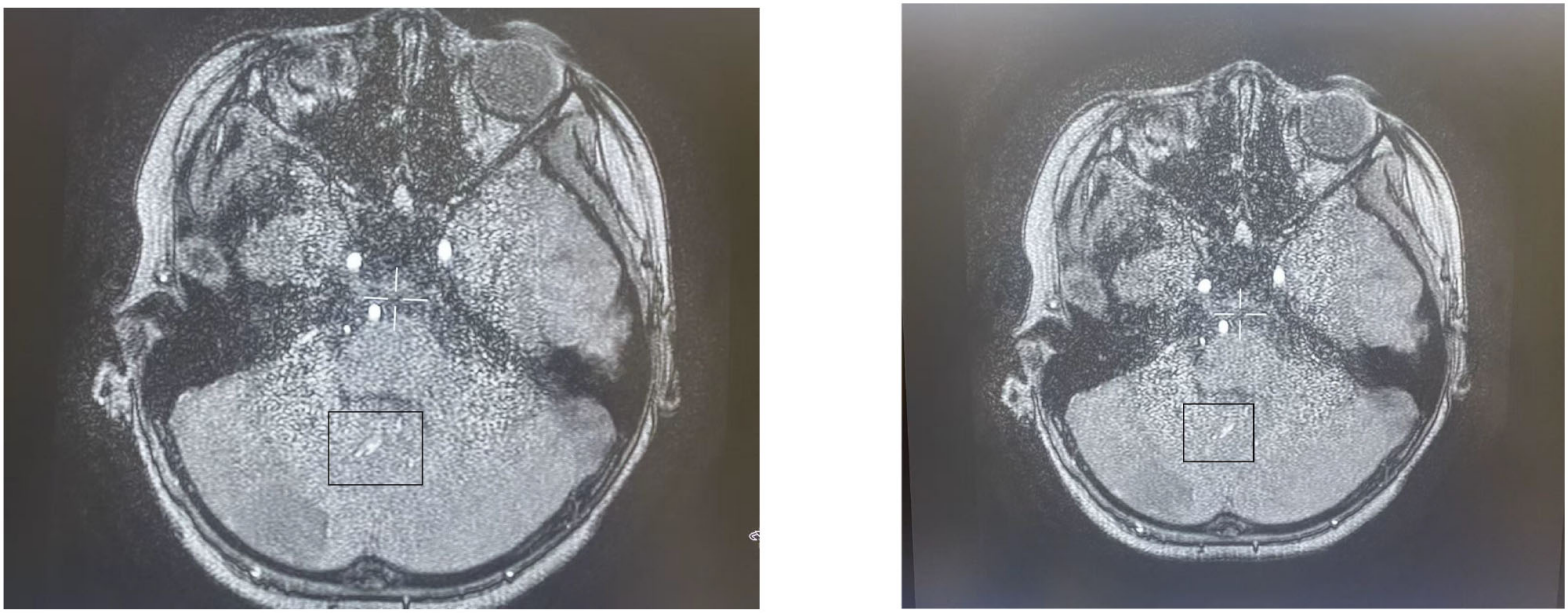
MRA performed on September 11,2024, showed slightly roughened posterior cerebellar artery walls with low-signal foci and potential small thrombi.

Upon admission to our department, the child’s external hospital cranial CT scan revealed atlantoaxial subluxation. Although the patient had a history of outdoor lake recreation, there was no head or neck injury, and the outdoor activity occurred over a month prior to the onset of symptoms. The child reported no discomfort during this period. Therefore, the atlantoaxial subluxation may have resulted from secondary factors such as cerebral herniation traction, cervical muscle spasms, or cranio-cervical tension imbalance. Additionally, atlantoaxial subluxation typically compresses vertebral arteries. The cerebral angiography at admission showed normal visualization of both vertebral arteries without stenosis or occlusion, indicating insufficient evidence for arterial compression. Further examinations were conducted. On September 9, 2024, ophthalmic fundus examination revealed optic disc edema with hemorrhage and tortuous dilation of blood vessels. Subsequently, cranial MRA (**Figure 5**) performed on September 11,2024, showed slightly roughened posterior cerebellar artery walls with low-signal foci and potential small thrombi. Cranial MRV demonstrated indistinct visualization of the superior sagittal sinus and bilateral transverse sinuses, with no significant abnormalities in other venous sinuses. Echocardiography revealed left coronary artery stenosis (upper limit of diameter: 0.35 cm, Z-value: 1.54). The child initially presented with cerebellar infarction, though prior to onset, there was a history of asthma and recurrent rashes on both lower limbs. During the course of the disease, elevated eosinophil counts were observed in both peripheral blood and bone marrow. Notably, the child had no head or neck trauma in the week preceding the onset. Neither cerebral angiography nor cranial MRA revealed any signs of arterial dissection, including endarterosal flaps, double lumen signs, false lumen formation, or intimal tears. Therefore, the diagnosis of arterial dissection lacked sufficient evidence.

**Figure 6:**
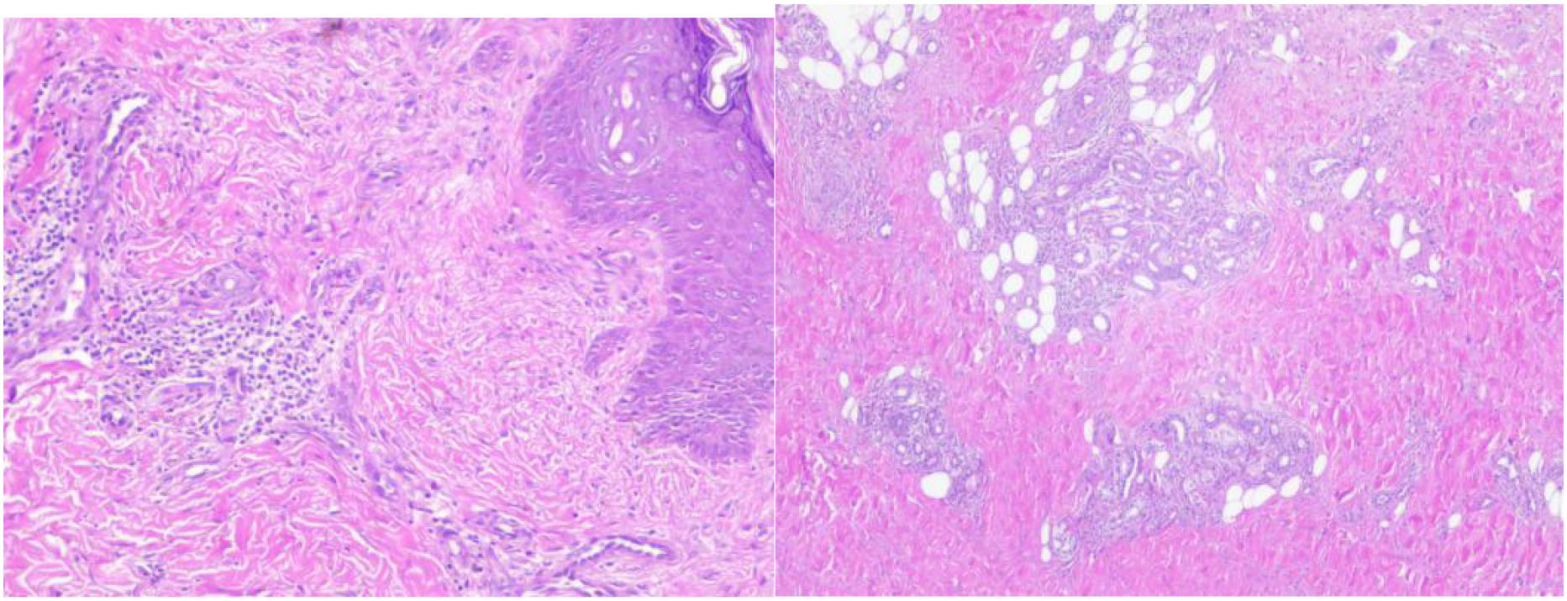
Skin pathological biopsy (Sudanine eosin staining). There were many lymphocytes and a few neutrophils infiltrating around the small blood vessels and accessories of the epidermis, and a few nuclear debris could be seen. Some vascular endothelium was damaged.

The patient presented with acute cerebella infarction at a young age, presenting neurological symptoms including dizziness, vomiting, and consciousness impairment. Physical examination revealed vasculitis-like skin rashes on both lower limbs accompanied by asymmetric limb weakness. Blood tests showed hyper eosinophilic syndrome and elevated IgE levels, while bone marrow cytology demonstrated increased eosinophils. Cranial MRI indicated cerebral infarction, brain herniation, and suspected thrombosis. Given the involvement of central nervous system, skin, and hematological systems, a definitive diagnosis required further evaluation through skin biopsy (lower limb rash, **Figure 6**). Histopathological analysis revealed abundant lymphocyte infiltration around dermal small vessels and appendages, with minimal neutrophil infiltration. Nuclear debris and partial vascular endothelial damage were observed, particularly giant cell reactions in the deep dermis of the left lower limb, consistent with vasculitis. These findings suggest that the infarct-like manifestations in the large vessel supply areas (cerebellar infarction + brain herniation) may be caused by EGPA-related vasculitis or microthrombosis. To further exclude central nervous system infection, a lumbar puncture was performed on September 14,2024, with stable vital signs, symmetrical pupils, and absence of herniation or intracranial hypertension. Routine cerebrospinal fluid analysis, biochemical tests, and cultures showed no abnormalities. Based on medical history, clinical presentation, and ancillary tests, the diagnosis aligns with the 2022 American College of Rheumatology (ACR) and European League of Associations for Rheumatism (EULAR) EGPA classification criteria for ANCA-associated vasculitis (4). Key features included a history of asthma attacks (+3 points), hospitalization with eosinophil counts reaching 4.9×10^9/L (≥5 points), and total score exceeding 8 points (>6 points). EGPA was confirmed after thorough exclusion of infections, tumors, diffuse connective tissue diseases, immunodeficiency disorders, and genetic metabolic disorders.

**Figure 7:**
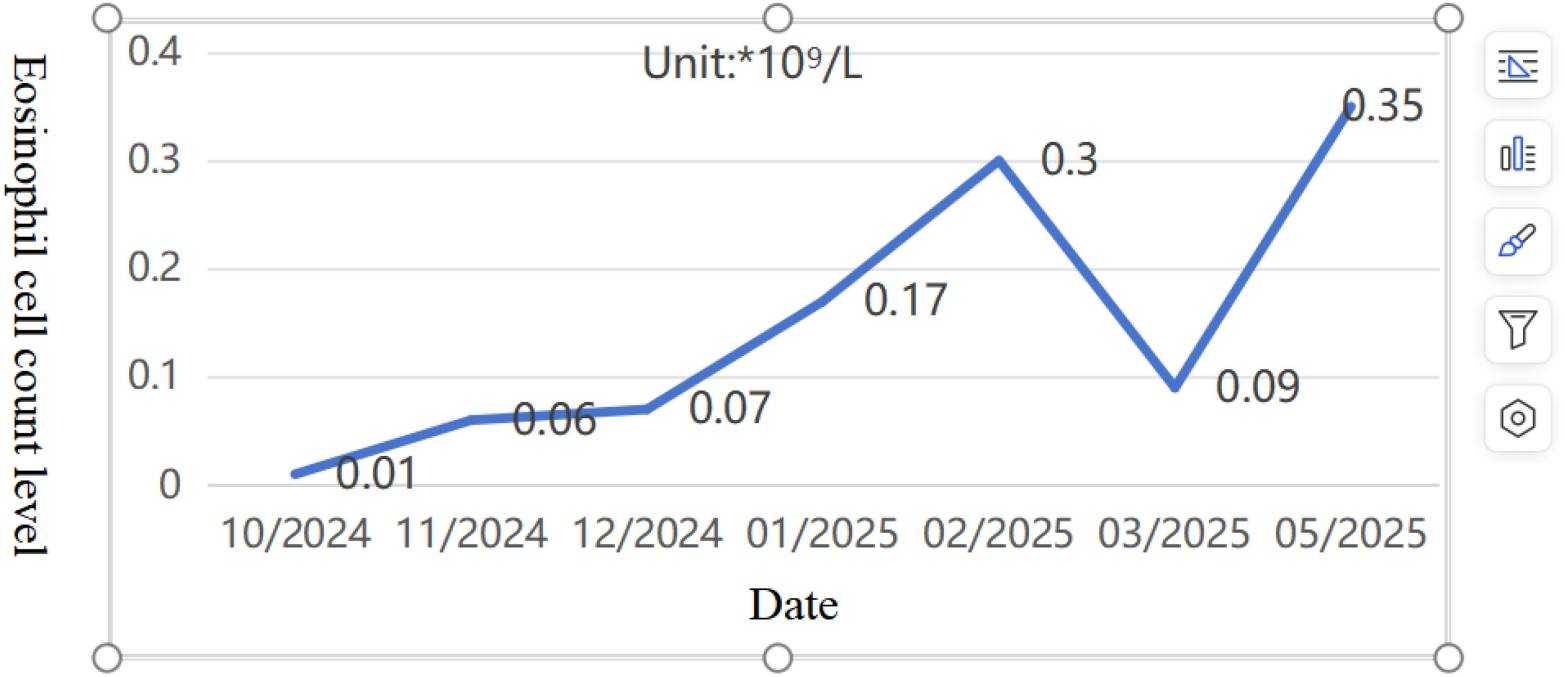
10/2024-05/2025 Eosinophil cell count level (Reference value 0.04-0.74*10^9^/L).

**Figure 8:**
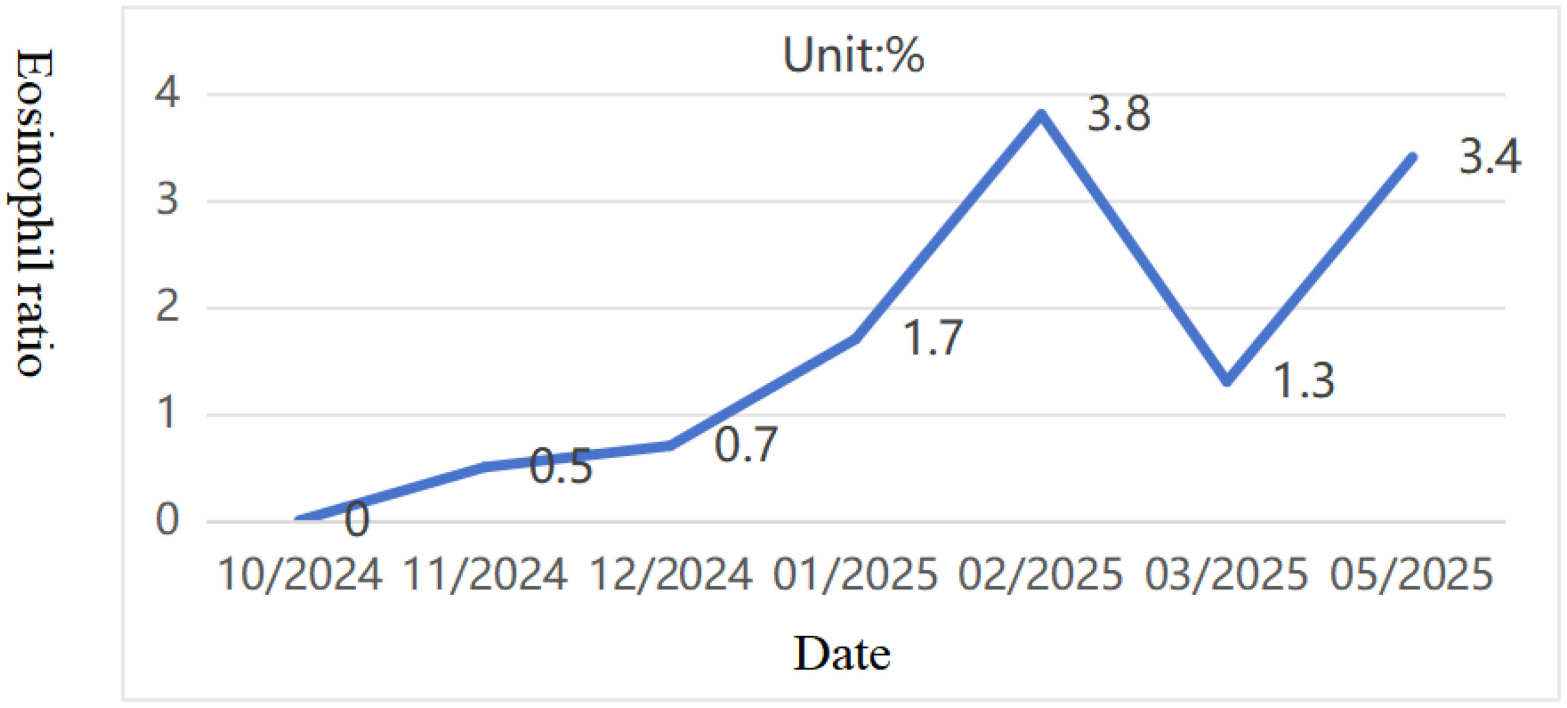
10/2024-05/2025 Eosinophil ratio (Reference value 0.5-9%).

Pediatric Vasculitis Activity Score (PVAS) evaluation: 1 point for persistent symptoms (including previous asthma attacks) + 19 points for new or worsening manifestations (4 points for ulcerated rash, 6 points for papilledema with hemorrhage, and 9 points for headache, altered consciousness, or cerebral infarction). On 09/09/2024, the diagnosis was confirmed, and the treatment regimen was adjusted to include adequate corticosteroids (48mg/day methylprednisolone). To reduce risks of microthrombosis or reinfarction, control vascular inflammation-induced thrombosis, and prevent short-term stroke recurrence, anticoagulation therapy was added with nadroparin calcium subcutaneous injection initially. On the fourth day of steroid administration (12/09/2024), blood tests showed white blood cell count at 16.61×10^9/L, eosinophil ratio 0.1%, and eosinophil count 0.01×10^9/L. Subsequently, subcutaneous sodium heparin calcium was switched to oral rivaroxaban anticoagulation. After excluding contraindications, CTX0.5g monthly infusion therapy was initiated on September 16. Hormone replacement therapy was gradually increased to oral prednisone 60 mg/day. The child’s condition progressively improved, muscle strength normalized, and independent walking resumed with slight instability. A follow-up cranial MRI on September 19th revealed abnormal signals in the right cerebellar hemisphere with partial softening foci, full supratentorial ventricles, tortuous posterior inferior cerebellar artery with hypoechoic areas on MRA, and localized hypoechoic changes in the right transverse and rectal sinuses on MRV. Prednisone was reduced to 50 mg/day after discharge. As of 02/02/2025, the patient received CTX 0.5g/month for 6 times (total dose 100mg/kg). Follow-up cranial MRI with MRA/MRV and DWI sequences revealed abnormal signals in the right cerebellar hemisphere with partial softening foci formation. The supratentorial ventricles were full, with tortuous and poorly defined posterior inferior cerebellar artery on the right side, along with localized hypoechoic areas in the right transverse sinus and straight sinus. Chest CT showed no parenchymal lesions. The PVAS score was 4 (1 for rash + 3 for cranial imaging changes), followed by a continued oral mycophenolate mofetil treatment on 16/02/2025. The last follow-up on 04/05/2025, showed no new rashes, normal limb muscle strength, no dizziness or consciousness impairment, and normal limb mobility. Eosinophil ratio was 3.4% with eosinophil count 0.35×10^9/L (**Figures 7**, **8**). Given the patient’s central organ involvement at onset, a higher recurrence risk was considered. Therefore, rituximab infusion (375mg/m² every six months) was added for maintenance therapy starting May 6th. Current oral medication regimen includes: Mycophenolate Mofetil capsules (0.5g morning, 0.375g evening), oral prednisone (5mg/day, reduced from May 7th), oral rivaroxaban anticoagulation, and calcium/vitamin D supplementation.

## 4 Discussion

First identified and documented in 1951 by pathologists Churg and Strauss, EGPA was initially named Churg-Strauss syndrome before being renamed EGPA in 2012. This rare condition is a type of anti-neutrophil cytoplasmic antibody (ANCA)-associated systemic vasculitis (AAV), primarily affecting small and medium blood vessels. It can occur at any age, with the median onset age ranging from 38 to 54 years (5). Studies indicate an estimated prevalence of 10.7~17.8/million cases per year, with 0.9~2.4/million new cases emerging annually. The disease shows no significant gender differences, familial clustering, or ethnic predisposition (6). Due to its low incidence, insidious onset, and diverse clinical manifestations, EGPA is frequently misdiagnosed or overlooked.

Current understanding indicates that the pathogenesis of EGPA primarily involves ANCA-mediated vascular wall damage and eosinophilic tissue infiltration, along with degranulation-induced injury. Based on ANCA positivity status, EGPA can be classified into two subtypes: vasculitis-type and non-vasculitis-type. The vasculitis-type subtype involves ANCA-induced neutrophil activation and degranulation, leading to endothelial cell damage, primarily manifesting as small vessel inflammation. The non-vasculitis-type subtype shows ANCA-negative status with eosinophils as the primary pathogenic factor. Activated eosinophils release large amounts of eosinophilic granuloproteins, various cytokines, and toxins that cause tissue damage, with pathological features mainly showing eosinophilic tissue infiltration (7). Studies indicate that ANCA-positive EGPA patients exhibit more vasculitis characteristics, making them more prone to peripheral neuropathy, renal involvement, and purpura (8, 9), while ANCA-negative patients show eosinophilic infiltration characteristics, increasing susceptibility to pulmonary infiltration and cardiac involvement (10). Cardiac involvement remains a high-risk factor for EGPA prognosis, typically presenting as myocarditis and coronary arteritis, rarely affecting large or medium arteries, and is the primary cause of mortality (11, 12). In this pediatric case, although ANCA-negative, vasculitis manifestations such as skin rash and central nervous system involvement were observed, accompanied by cardiac involvement. Cardiac color Doppler ultrasound monitoring during the course of the disease showed high-limits coronary artery diameter. After two months of standardized treatment, follow-up examinations revealed normalized coronary arteries. As for pulmonary infiltration, no signs such as ground-glass opacities, consolidation, linear opacities, nodules, bronchiectasis, or pleural effusion were detected in chest CT scans during follow-up. In a retrospective analysis of pediatric cases of EGPA in the United Kingdom, no children tested positive for ANCA. Given the limited number of pediatric cases of EGPA, it is difficult to determine whether there is a correlation between clinical manifestations and pediatric ANCA (13).

The clinical course of EGPA is generally divided into three phases: (1) Prodromal phase: Characterized by respiratory hypersensitivity, such as allergic rhinitis and asthma, often accompanied by nasal polyps and sinusitis; (2) Eosinophilic infiltration phase: Marked by eosinophilic infiltration in affected organs and granuloma formation; (3) Vasculitis phase: May involve tissues and organs such as the lungs, nervous system, skin, gastrointestinal tract, heart, and kidneys. These phases may occur independently or overlap. Due to multi-system involvement, clinical presentations vary widely in initial symptoms and can present with distinct or overlapping manifestations during disease progression. A study showed that (9)55.1% of EGPA patients experienced neurological involvement, predominantly peripheral nervous system involvement with relatively rare central nervous system involvement. In this case, early follow-up fundus examination revealed bilateral optic disc edema with hemorrhage. Although intracranial hypertension caused by cerebral herniation during the initial stage could compress the optic nerve, EGPA-induced optic nerve involvement cannot be ruled out. Most EGPA patients present with respiratory symptoms as initial manifestations, while neurological symptoms at first diagnosis are extremely rare, particularly in children. Using search terms “eosinophilic granulomatous polyangiitis”, “central nervous system”, and “children” in CNKI and Wanfang databases yielded no pediatric reports, with all documented cases being adults. Central nervous system involvement predominantly manifests as cerebral infarction, cerebral hemorrhage, or subarachnoid hemorrhage, with rare cases involving central demyelinating lesions, meningitis, or spinal cord abnormalities (14, 15). When performing a PubMed search using the same keywords, Example 1 was identified: A 10-year-old girl with a history of recurrent cough and asthma complicated by peripheral blood eosinophilia was admitted to the hospital presenting with maculopapular rashes on her hands and feet, lung rales, laboratory findings showing elevated eosinophil count, positive anti-myelin peroxidase antibody (MPO-ANCA) test, and immunoglobulin E levels>2500 IU/mL. Imaging revealed multiple patchy and nodular high-density shadows in both lungs along with sinusitis. Bone marrow cytology demonstrated significantly increased eosinophils, while skin biopsy confirmed leukocytoclastic vasculitis. During hospitalization, the child developed seizures. Cranial MRI showed multiple abnormal signal shadows in bilateral cerebral cortex, and EEG results indicated epileptic activity. After administering methylprednisolone pulse therapy combined with cyclophosphamide, the patient’s cough and asthma symptoms subsided, the rash resolved, and no recurrence of seizures was observed (16). When removing the “central nervous system” filter and expanding the search scope using keywords “eosinophilic granulomatous polyangiitis” and “pediatric” in PubMed database, two cases with concurrent central nervous system involvement were identified: A 14-year-old male presented with prolonged fever, weight loss, myalgia, arthralgia, and purpuric rash, followed by finger numbness, sinusitis, testicular pain, pulmonary infiltrates, asthma, and pericardial effusion. Laboratory tests showed eosinophils accounting for 58% of peripheral blood cells, while skin biopsy revealed necrolytic vasculitis with eosinophilic infiltration. Although initial treatment with glucocorticoids and cyclophosphamide improved the child’s condition, subsequent cerebral vasculitis secondary to epilepsy led to hypoxemia and cardiac arrest resulting in death (17). The patient had recurrent bilateral lower limb rashes for one year prior to onset and a history of asthma six months earlier. Presenting with dizziness, vomiting, and altered consciousness, laboratory tests showed elevated eosinophils in peripheral blood and bone marrow. Cranial MRI revealed abnormal signals in the right cerebellar hemisphere, suggesting cerebral infarction complicated by cerebellar tonsillar herniation. Pathological skin biopsy confirmed vascular endothelial damage. Based on clinical presentation and laboratory findings, the diagnosis of Evansian glomerulonephritis (EGPA) was established after thorough exclusion of other causes. The diverse neurological manifestations observed in EGPA patients underscore the critical importance of thorough medical history evaluation and laboratory findings during clinical practice. Patients with a history of allergic conditions (e.g., asthma, allergic rhinitis, urticaria) or elevated eosinophil levels should be highly suspected for EGPA. Notably, recent studies recommend excluding EGPA in all asthma patients presenting with neurological symptoms (11).

In clinical practice, the five-factor score (FFS) developed by the French Vasculitis Research Organization is commonly used to evaluate risk factors and prognosis for EGPA. The criteria include: 1) gastrointestinal involvement; 2) cardiac involvement; 3) serum creatinine>150 μmol/L; 4) age>65 years; 5) absence of otolaryngological involvement. Each criterion is scored as 1 point, with higher scores indicating poorer prognosis (18). For EGPA treatment, it is recommended to follow the traditional vasculitis management protocol, where systemic corticosteroids (GC) serve as the cornerstone therapy. Studies indicate that in FFS = 0 cases, monotherapy with corticosteroids typically resolves rash, allergic rhinitis, and pulmonary infiltration within one week. For patients with poor corticosteroid response, combination therapy with moderate-intensity immunosuppressants such as azathioprine or methotrexate is recommended. For FFS≥1 cases, a 6–12 month combination of corticosteroids and immunosuppressants is advised, with severe cases requiring pulse steroid therapy (19).

In recent years, new therapeutic options for EGPA have been continuously emerging, with the introduction of biologics expanding treatment approaches. Based on their mechanisms of action, current EGPA-related drugs can be categorized into two types: those targeting vasculitis and those addressing eosinophilia. 1) Vasculitis-targeting therapies: ANCA plays a significant role in AAV, and B cells serve as precursors to ANCA-producing plasma cells. Rituximab (RTX), a chimeric monoclonal antibody targeting B-cell CD20 antigens and inducing B cell depletion, has shown clinical efficacy and safety in both induction and maintenance phases for GPA and MPA (20). The growing experience in AAV applications has also expanded RTX’s use in EGPA. However, experts from the French Vasculitis Study Group (FVSG) do not recommend RTX as first-line induction therapy for EGPA, but suggest it as a second-line or follow-up treatment in severe refractory or relapsed cases, particularly after cyclophosphamide failure (21). The American College of Rheumatology (ACR) recommends cyclophosphamide or rituximab for active severe EGPA patients, while suggesting methotrexate, azathioprine, or mycophenolate mofetil over rituximab for maintenance during remission phases (22). Additionally, cyclophosphamide demonstrates greater efficacy than rituximab in crossing the blood-brain barrier (23). 2) Treatment for Eosinophilia: Interleukin-5 (IL-5), a key cytokine driving eosinophil maturation and proliferation, can be inhibited by the monoclonal antibody mabthera (mabthera) through blocking IL-5-receptor binding. In 2010, two clinical studies in EGPA patients provided the first evidence of mabthera’s efficacy (24). The American College of Rheumatology (ACR) recommends initiating mabthera with glucocorticoids (GC) over methotrexate, azathioprine, or mycophenolate mofetil and GC for active, non-severe EGPA patients. This case presents with central nervous system symptoms as initial manifestations, likely indicating cerebral infarction complicated by brain herniation. Given the severity of the condition, cyclophosphamide combined with GC was selected for disease induction remission therapy. After six cyclophosphamide pulse courses, eosinophil levels remained normal without recurrent rashes, asthma attacks, or psychiatric changes. Follow-up cranial MRI after six months showed softening lesions without new findings. The patient tested negative for ANCA, with laboratory tests initially showing elevated eosinophils. While mabthera could be considered, its high cost and lack of established safety and efficacy in pediatric populations (especially under 12 years old) led us to prioritize other treatment options after thorough safety evaluations and parental consultation.

In both the combined case and this individual case, central nervous system involvement was observed with clinical manifestations including seizures, epilepsy, finger numbness, and cerebral infarction. All three patients received cyclophosphamide induction therapy. Case 2 showed symptom improvement with initial cyclophosphamide and corticosteroid combination therapy, but developed secondary cerebrovascular vasculitis during disease progression, ultimately leading to hypoxemia and cardiac arrest resulting in death. Cases 1 and this child demonstrated relatively successful treatment outcomes (16, 17). We reviewed a case report of an adult EGPA with central nervous system involvement (25): A 60-year-old female patient presented with fever, chills, muscle pain, and limb numbness. Laboratory tests revealed eosinophilia in peripheral blood smears and positive perinuclear ANCA (P-ANCA). Renal biopsy showed eosinophilic infiltration in glomeruli and renal interstitium, while cranial MRI identified compression lesions in the left corpus callosum and periventricular congestion. The diagnosis was eosinophilic granulomatous polyangiitis with peripheral and central nervous system involvement. Treatment with corticosteroids combined with cyclophosphamide led to rapid symptom improvement. These findings suggest that combined hormone and cyclophosphamide pulse induction therapy is effective for EGPA with central nervous system involvement.

This case report details a patient who completed cyclophosphamide induction therapy followed by oral mycophenolate mofetil capsules. Given the involvement of vital central organs at onset and the high risk of recurrence, we considered adding biologic therapy. While pembrolizumab was an option, its high cost and unestablished safety in pediatric patients (particularly under 12 years) led to parental concerns, resulting in its exclusion. Studies have shown that rituximab administration in EGPA reduces IL-5 production, likely through suppression of B-T cell interactions (26). A retrospective European collaborative study demonstrated rituximab’s efficacy in treating EGPA vasculitis recurrence, with no significant difference between ANCA-positive and ANCA-negative patients (27). The first rituximab maintenance treatment was administered on 04/05/2025. Short-term follow-up demonstrated stable disease progression with no new neurological manifestations or abnormal findings identified through imaging evaluations. However, given the potential for recurrent and unpredictable central nervous system involvement in EGPA, long-term follow-up data will be required to assess the efficacy and safety thresholds of this treatment regimen, thereby providing evidence-based support for improving quality of life in children with such rare diseases.

## Data Availability

The original contributions presented in the study are included in the article/supplementary material. Further inquiries can be directed to the corresponding author.
